# Evaluating evidence-based health information from generative AI using a cross-sectional study with laypeople seeking screening information

**DOI:** 10.1038/s41746-025-01752-6

**Published:** 2025-06-09

**Authors:** Felix G. Rebitschek, Alessandra Carella, Silja Kohlrausch-Pazin, Michael Zitzmann, Anke Steckelberg, Christoph Wilhelm

**Affiliations:** 1 Harding Center for Risk Literacy, Faculty of Health Sciences Brandenburg, University of Potsdam, Potsdam, Germany; 2 Max Planck Institute for Human Development, Berlin, Germany; 3 Department of Developmental and Social Psychology, University of Padova, Padova, Italy; 4 Institute of Health, Midwifery and Nursing Science, Medical Faculty, Martin Luther University Halle-Wittenberg, Halle (Saale), Germany; 5 International Graduate Academy (InGrA), Institute of Health, Midwifery and Nursing Science, Medical Faculty, Martin Luther University Halle-Wittenberg, Halle (Saale), Germany

**Keywords:** Education, Breast cancer, Cancer screening, Prostate cancer, Health policy, Health services, Public health, Decision making

## Abstract

Large language models (LLMs) are used to seek health information. Guidelines for evidence-based health communication require the presentation of the best available evidence to support informed decision-making. We investigate the prompt-dependent guideline compliance of LLMs and evaluate a minimal behavioural intervention for boosting laypeople’s prompting. Study 1 systematically varied prompt informedness, topic, and LLMs to evaluate compliance. Study 2 randomized 300 participants to three LLMs under standard or boosted prompting conditions. Blinded raters assessed LLM response with two instruments. Study 1 found that LLMs failed evidence-based health communication standards. The quality of responses was found to be contingent upon prompt informedness. Study 2 revealed that laypeople frequently generated poor-quality responses. The simple boost improved response quality, though it remained below required standards. These findings underscore the inadequacy of LLMs as a standalone health communication tool. Integrating LLMs with evidence-based frameworks, enhancing their reasoning and interfaces, and teaching prompting are essential. Study Registration: German Clinical Trials Register (DRKS) (Reg. No.: DRKS00035228, registered on 15 October 2024).

## Introduction

The internet has become a primary source of health information for people in Western countries^1^. However, many online resources fail to adhere to evidence-based health communication standards^2^. As the practical implementation of guidelines for evidence-based health communication^3,4^ remains challenging, much of the health information available online does not support informed health decision-making, and it lacks necessary accuracy and rigor, and in some cases even spreads misinformation, particularly in areas such as cancer^5^. The rapid emergence of artificial intelligence (AI) tools, particularly large language models (LLMs) such as OpenAI’s ChatGPT, Mistral AI’s Le Chat, has opened novel possibilities for digital health communication – even in professional settings^6^. Laypeople are increasingly turning to these platforms to seek answers to health-related questions^7,8^. However, can LLM users reliably obtain correct information that complies with established health communication guidelines? To address this, we conduct two complementary studies aimed at evaluating and enhancing the quality of health communication provided by LLMs.

Although LLMs are not designed for health communication, laypeople use them to gather health information. In a convenience online sample of adult panellists from February to March 2023, 7.2% reported regularly using LLMs for health-related topics—often in combination with search engines and online health communities^9^. This number may have increased, as indicated by studies with varying methodologies (e.g. assessment of frequency of use) and populations (e.g. up to 79% among LLM users^8^): 21.2% of an online community convenience sample in May 2024^10^, 20.3% of an online representative sample for Germany in a preprint^11^, and 32.6% of a U.S. online panel in February 2024 reported using LLMs for health information^12^.

Though often correct, LLM-generated responses to health-related queries show substantial variability^13,14^. The ‘accuracy’ or ‘reliability’ of LLM responses has been subject to numerous descriptive studies, including a recent systematic review of 88 patient education studies^15^. Yet, what does accuracy or reliability truly mean in this context? LLM-generated responses to health-related questions have been assessed in various ways, such as readability^16–21^, expert-rated appropriateness of factual explanations^18,21–30^, and agreement with health authorities and guidelines^31–38^. However, what remains missing, is the use of the best available evidence as the ground truth.

LLM information and communication studies consistently lack a well-defined healthcare premise (e.g. informed decision-making). This absence undermines both the criteria for response assessment (e.g. compliance with evidence-based health communication standards) and the structured selection of prompts (e.g. aligning with information requirements). Moreover, these studies often fail to ground their assessments in the best available evidence, such as high-quality evidence synthesis. Evidence-based guidelines, such as those developed by the Working Group for Good Practice in Health Information (GPHI), provide a structured framework for health communication^4,39^. They emphasize, for instance, the importance of using numerical data to present risks and benefits based on the best available evidence. Regarding validated instruments for assessing information quality, at least three LLM studies^13,16,40^ have applied the DISCERN standards, which from today’s perspective, are considered outdated, while one study utilized a validated patient information quality instrument^41^.

The primary aim of Study 1, therefore, is to assess the extent to which content generated by state-of-the-art LLMs—specifically OpenAI’s ChatGPT, Google Gemini, and Mistral AI—adheres to guideline-based criteria, with a particular focus on essential aspects of health risk communication for informed decision-making. These criteria encompass clear communication of benefits and harms, inclusion of reference class information, the appropriate presentation of numerical effects, the quality of evidence, and accurate interpretation of screening results. Additionally, they cover background information, such as proper referencing and disclosures of conflicts of interest. The first research question (RQ) directly asks: Does the health risk communication provided by LLMs reflect the best available evidence? The hypothesis is that LLMs largely fail to meet standard criteria for evidence-based health risk communication, as indicated by more than 50% of responses deviating, on average, from these guidelines. Using both MAPPinfo, an established assessment instrument^42^, and ebmNucleus, an assessment proposal derived from the Guideline Evidence-Based Health Communication^3^, Study 1 systematically examines the quality of outputs generated in response to repeated prompts concerning mammography for breast cancer screening (BC) and the PSA test for prostate cancer screening (PC).

Prompt engineering refers to the effective interaction with LLMs to obtain optimal responses. The effect of prompt quality on LLM-generated responses has been demonstrated, for instance, in a March 2023 study on cardiovascular health advice, which used a non-validated expert-rating system to assess correctness, including the dimension of simplification in presentation^43^. However, it remains unknown whether prompt engineering also leads to more evidence-based responses for health decision-making. Therefore, Study 1 explores whether more informed prompts containing context knowledge increase LLM response quality. Accordingly, our RQ2 asks: Does a systematic variation of the contextual informedness of prompting increase the proportion of evidence-based LLM responses? We assume at least a moderately strong association between the grade of prompting informedness and the extent to which LLM responses are scored as evidence-based.

We expect that our findings from this systematic variation of LLM prompting will align with prior studies that have used different accuracy benchmarks. Building on this, we aim to provide causal evidence that supporting laypeople in prompting can improve the quality of LLM responses they receive. However, since little is known about how effectively laypeople prompt without guidance, Study 2 first describes their LLM performance by assessing the compliance of LLM outputs with evidence-based health communication standards—using the same instruments and conditions as in Study 1. Second, earlier studies have shown that prompt engineering training can improve response quality, as demonstrated, for example, with journalists^44^. However, training programs face hurdles in practical implementation, such as time requirements, costs, and the need for human involvement. Beyond traditional education, boosting^45^ could be a more feasible approach. Boosts–interventions that modify human cognition or the environment using behavioral insights to enhance people’s competences–have been proposed for navigating digital information architectures, including applications in fake news detection^46^ and health information search^47^. Study 2 examines RQ3: Does a minimal boosting intervention that encourages laypeople to provide more informed prompts increase the proportion of evidence-based responses?

To sum up, we aim to describe the limitations of LLM with regard to the standards of evidence-based health communication, demonstrate the potential for improvement through contextually more informed prompting, confirm the expected shortcomings of human prompting, and provide evidence that a simple intervention can help laypeople obtain more evidence-based health information. A study protocol was published in advance^48^.

## Results

### Study 1

**Fig. 1:**
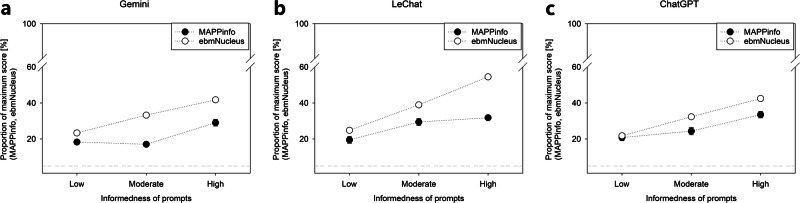
Results of study 1. The dependence of information quality as elicited with the help of two metrics (MAPPinfo and ebmNucleus) on the informedness of prompts is demonstrated across Gemini (**a**), Le Chat (**b**), and ChatGPT (**c**). Error bars show the standard error of the average rate of fulfilled criteria across trials.

LLMs’ responses had a median length of 211 (IQR: 158–278) words per prompt. All LLMs provided a response to every prompt. Our systematic variation of prompt informedness revealed the expected pattern across the tested LLMs (Fig. 1a–c), demonstrating that more informed prompting led to higher scores according to both assessment schemes MAPPinfo (*F*(2,351) = 40.25, *p* < 0.001, ${\eta }_{{\rm{p}}}^{2}=0.19$) and ebmNucleus (*F*(2,351) = 528.41, *p* < 0.001, ${\eta }_{{\rm{p}}}^{2}=0.75$). The effect of informedness varied depending on the LLM as indicated by interactions for MAPPinfo (*F*(4,351) = 3.48, *p* = 0.008, ${\eta }_{{\rm{p}}}^{2}=0.04$) and ebmNucleus (*F*(4,351) = 12.11, *p* < 0.001, ${\eta }_{{\rm{p}}}^{2}=0.12$).

### Study 2

The sample (Table 1) consisted of 52.3% female, without any non-binary participants. Age ranged from 19 to 78 years, with a mean age of 46.4 years (SD = 15.5). The majority of participants were highly educated: 16.3% held a Master’s degree, 43.3% a Bachelor’s degree, and 4.3% a PhD. Additionally, 20.7% had completed school education up to the age of 18, while 9.7% had obtained professional or technical qualifications. A small proportion (5.7%) reported having no formal education beyond the age of 16. A total of 63.0% of participants reported having at least some experience with LLMs, and notably, 31.7% used them at least once per month to seek health information. On average, participants submitted 3.0 prompts (SD = 1.0). Those prompts had a median length of seven words (IQR: 5–9). All LLMs generated a response for every prompt, and the intervention increased the prompt word count (*p* = 0.032). The LLMs’ responses to these prompts had a median length of 188 words (IQR: 126–254) per response.

**Table 1:** Sample description according to intervention condition

	Intervention	Control	Difference (p)
Gender (% female)	53.7	51.0	–
Age in years (M[SD])	46.5 [15.8]	46.3 [15.3]	–
Education			–
PhD (%)	5.4	3.3	
Master’s degree (%)	18.1	14.6	
Bachelor’s degree (%)	39.6	47.0	
School education up to age 18 (%)	18.8	22.5	
Professional or technical qualifications (%)	12.1	7.3	
No formal education (%)	6.0	5.3	
At least some experience with LLM (%)	65.8	60.3	–
At least once per month LLM health info. (%)	30.2	33.1	–
Preferred topic			–
Breast cancer screening (%)	55.0	52.3	
Prostate cancer screening (%)	45.0	47.7	
Number of prompts out of four (M[SD])	3.0 [1.0]	2.9 [1.0]	–
Prompt word count (M[SD])	8.2 [6.4]	7.3 [3.4]	0.032

Independent of the specific LLM, the information elicited in the control condition reached only approximately 17% of the possible maximum score in MAPPinfo and 13% in ebmNucleus. A regression analysis of the control condition (*F*(6,144) = 7.75, *p* < 0.001) revealed that participants generated higher quality information (ebmNucleus) when they had a higher level of education (*β*_standardized_ = 0.16, *p* = 0.032) and more experience with LLMs (*β* = 0.26, *p* = 0.007).However, more frequent LLM usage for seeking health information was negatively associated with information quality (*β* = −0.25, *p* = 0.009). Neither age (*p* = 0.219) nor gender (*p* = 0.952) had a significant influence, when controlling for the chosen topic, with prostate cancer screening prompts yielding higher-quality information (*β*_standardized_ = 0.41, *p* = 0.003).

**Fig. 2:**
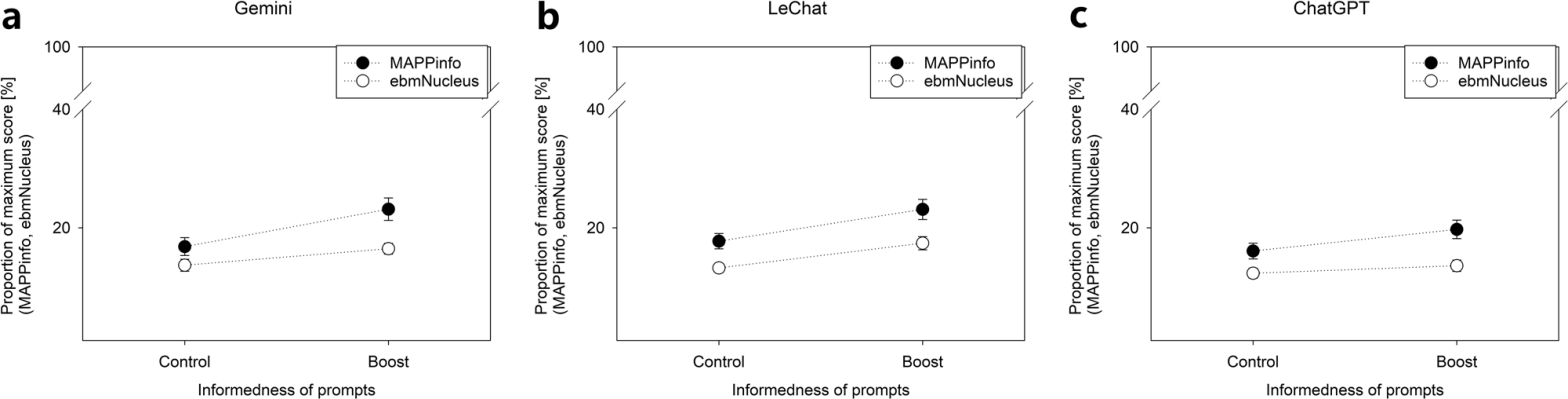
Results of study 2. The improvement of information quality as elicited with the help of two metrics (MAPPinfo and ebmNucleus) with the help of a boost is demonstrated across Gemini (**a**), Le Chat (**b**), and ChatGPT (**c**). Error bars show the standard error of the average rate of fulfilled criteria across participants.

The boosting intervention for more informed prompting moderately improved the quality of elicited health information across all LLMs (Fig. 2a–c), as indicated by ANOVA main effects for MAPPinfo (*F*(1,294) = 15.26, *p* < 0.001, ${\eta }_{{\rm{p}}}^{2}=0.05$) and ebmNucleus (*F*(1,294) = 12.12, *p* = 0.001, ${\eta }_{{\rm{p}}}^{2}=0.04$). The effect of the intervention neither varied for MAPPinfo (*F*(2,294) = 0.32, *p* = 0.726) nor for ebmNucleus (*F*(2,294) = 1.18, *p* = 0.307) across the LLMs.

## Discussion

Our findings highlight several key insights into the quality of health information provided by LLMs and the role of prompt-informedness in improving response quality.

First, our findings reinforce previous concerns that LLMs are not designed to inform health decision making and, in particular, they do not comply with the standards of evidence-based health information. Even when well-informed prompts were crafted by researchers—ensuring maximum accordance with information requirements—not even 50% of maximum compliance could be reached with ChatGPT and Gemini responses (somewhat better in Le Chat).

Second, our results confirm that the quality of elicited information directly depends on prompt informedness. This was systematically demonstrated by the gradual prompt manipulation, which aligned with the information requirements for informed decision-making. Importantly, this relationship was observed across both assessment tools—ebmNucleus, which explicitly focuses on these information requirements, and MAPPinfo, an established but broader quality assessment instrument.

Third and most notably, we provide causal evidence that a simple boosting intervention—reminding users to inquire about possible consequences of medical options—improved the quality of LLM-generated health information. This extends previous research on digital navigation boosts^45,47^, demonstrating that those behavioral interventions can help users seek better information. Moreover, our boost could be easily implemented at LLM interfaces. Theoretically, a hybrid approach would also be conceivable, in which evidence-based health communication prompting structures like the OARS rule are not only shown to users but also embedded into system-side chain-of-thought prompts^49^. This could benefit users who do not engage with optional guidance. However, such integration should be designed carefully, as fully automated scaffolding may reduce users’ awareness and active involvement in their own decision-making, particularly in health contexts that require personal judgment and reflection.

These insights may also inform the development of guidelines for responsible LLM health communication, which should inform about health prompt engineering, the potential of simple user-side prompting interventions, and standards and tools for output quality assessments, such as MAPPinfo and ebmNucleus.

Despite the strengths of our study, several limitations warrant consideration. The relatively low scores obtained with MAPPinfo may suggest that traditional health information assessment tools need to be carefully adapted for evaluating LLM outputs. However, this does not imply that the fundamental quality criteria for evidence-based health information should change. These criteria are derived from established evidence and codified in guidelines, forming the basis of our investigation. MAPPinfo was originally designed for structured health information sources, such as websites and brochures. While LLM-generated responses are dynamic and interactive, they should still adhere to the same evidence-based standards. If LLM-generated health information consistently fails to meet these standards, this raises concerns about their appropriateness as a source for informed medical decision-making rather than the validity of the assessment tools themselves.

The lower scores observed in participant-driven prompting with ebmNucleus may be explained by the task instructions. Participants were asked to seek general information about BC or PC screening, rather than specifically about mammography or PSA testing. This methodological choice influenced scoring outcomes because broad inquiries (e.g., “Tell me about breast cancer screening”) may generate responses covering multiple screening technologies, leading to less targeted benefit-harm presentations. While this directly affected only 8 out of 38 points in MAPPinfo, it influenced 18 out of 27 points in ebmNucleus, where a structured benefit-risk assessment is central (partial conceptual overlap is reflected in the limited convergent validity, for Study 1 with *r* = 0.45, for Study 2 with *r* = 0.31 in relation to the proportions of maximum scores). Nevertheless, these findings reflect real-world search behaviors, where users may not always phrase their questions in the most targeted manner.

Another limitation is that our Study 1 relied on single-turn prompts, rather than engaging in multi-turn conversations. This should ensure controlled and systematic prompt variations, making it easier to isolate the effects of prompt informedness. Given that conversational interactions with LLMs can improve information quality by allowing users to clarify, elaborate, or challenge information, future studies should explore the effect of conversation. In contrast, Study 2 provided a more naturalistic setting, enabling participants to engage in multi-turn conversations with the LLMs. Notably, these LLM conversations did not lead to responses of high quality in terms of evidence-based health communication. Future research could investigate how information quality evolves over time within a more extensive conversation.

Furthermore, it is possible that today’s LLMs may yield better evidence-based responses. However, there is currently no indication that LLMs are explicitly trained on evidence-based health communication principles. Additionally, while some newer models have real-time web access, this does not necessarily imply better compliance with EBM. Future studies should assess how real-time retrieval mechanisms influence response accuracy and bias in health-related LLM interactions.

Our study suggests that differences in education level and prior LLM experience influence the quality of information users retrieve. While more experienced users generated higher-quality responses, those who frequently used LLMs for health-related searches retrieved lower-quality information. This highlights a potential risk of digital inequality, where less-experienced users may unknowingly rely on incomplete or misleading AI-generated health information. Future research should investigate whether LLM interfaces could be adapted to mitigate such disparities, for example, by providing adaptive guidance based on user expertise levels.

LLMs generate output based on probabilistic language modeling, meaning they can reinforce existing biases in health communication. For instance, certain populations may receive different risk-benefit framing depending on how they phrase their queries. Given that health decisions disproportionately affect marginalized groups, fairness considerations in AI-generated health information should be systematically addressed. One possible approach is to audit LLM-generated health responses and related effects across demographic groups to detect and correct disparities. A prerequisite for this lies in future research that focuses on the impact of improved LLM-generated responses – how they shape users’ actual knowledge. Here it is important to distinguish studies that assess how well participants are actually prepared for making an informed decision from assessments of perceived informedness. Subjective impressions of being well-informed may not necessarily align with communication quality. In the case of intervention studies (e.g. boosting) this might promote misleading interpretations of effectiveness.

To sum up, LLMs are used as health information sources, yet our findings emphasize that they do not inform appropriately. While informedness plays a crucial role in improving response quality, even very informed prompts fail to ensure full compliance with standards of evidence-based health information. As LLMs continue to evolve, AI developers must focus not only on integrating the best available clinical evidence but also on leveraging behavioral science insights to optimize how information can be summarized and presented in balanced and transparent way^50^. Then can AI-driven health communication support knowledge acquisition, correct risk perceptions, and enable informed decision-making. But even in the light of further improvement, laypeople must also be informed about how well LLMs perform and where these models fall short, as users may hold unrealistic expectations about algorithm quality^51^.

Finally, as it is true for every algorithm system that is to be widely implemented in healthcare, the system’s stakeholders need to understand related consequences for different groups and stakeholders^52^; ideally with the help of large randomized trials.

## Methods

### Study 1

Study 1 employed systematic prompting variations to assess the compliance of LLM-generated health risk information with evidence-based health communication standards. Reporting followed the STROBE^53^ and TRIPOD-LLM^54^ guidelines.

### Design and data collection

This content analysis aimed to systematically evaluate the quality of responses generated by three LLMs—OpenAI’s ChatGPT (gpt-3.5-turbo), Google Gemini (1.5-Flash), and Mistral AI Le Chat (mistral-large-2402)—when prompted with health-related queries. These LLMs were selected based on their accessibility and public awareness in Germany during the summer 2024, as they were available without payment or the need to disclose personal data (except for an email address). At that time, specialized medical LLMs did not meet these criteria. The study followed a pre-registered content analysis design (AsPredicted, Registration No. 180732), and was conducted between June and July 2024. Since no human participants were involved and no personal data were used, ethical review was not required.

Our systematic prompting strategy (Table 2) included 18 prompts (Supplementary Tables 1 and 2) for both mammography and for the PSA test. The strategy varied prompts across three levels of contextual informedness: (1) highly informed prompts, which included technical keywords, (2) moderately informed prompts, which used more generic synonyms, and (3) low-informed prompts, which omitted key terms entirely. Furthermore, the strategy incorporated six information requirements that were chosen – in contrast to patients’ subjective information needs^55^ – because they are essential for deliberately weighing the probabilistic consequences of utilizing versus not utilizing a medical option (decision-analytic approach^56^), while taking also uncertainties and the special case of screening test-specific conditional probabilities (reliability of the option) into account (as intended by patient decision aids^57^). Fulfilling all selected requirements may enable an individual to make an informed medical decision^58^ – one of the key principles of evidence-based medicine.

**Table 2:** Prompting strategy underlying systematic testing

Information requirements	Low informed	Moderately informed	Highly informed
Listing possible benefits and harms	Asking for guidance	Asking for consequences	Asking for benefits and harms
Explaining single event probabilities	Asking for the chance	Asking for an explanation	Asking for reference conditions
Quantifying screening benefits	Asking for the advantage	Asking for a probability	Asking for an absolute effect
Quantifying numerical screening harms	Asking for the disadvantage	Asking for a probability	Asking for an absolute effect
Informing about evidence quality	Asking for an estimate	Asking for reliability	Asking for the study quality
Interpreting test result	Asking for positive result meaning	Asking for the positive predictive value	Providing input for conditional reasoning

LLMs are probabilistic models that produce variable outputs even when given the exact same prompt. To ensure a valid assessment—while many previous studies have relied on insufficient prompt repetitions (e.g., around 1–4)^14,31^ —we designed our study with a sample size of twenty independent trials per prompt to account for low probability responses. The three LLMs were accessed via API, without output length limitations, resulting in a total of 2160 prompts.

Mammography for BC and the PSA test for PC screening were selected as study topics because they represent two of the most common cancers worldwide, affecting millions of individuals and posing significant health implications for screening decisions. Both involve complex risk-benefit considerations in screening and treatment, require clear, evidence-based health communication to support informed decision-making. Additionally, BC and PC screenings have been subject to distorted public perceptions^59,60^, as well as ongoing debate due to concerns over overdiagnosis, false positives, and varying guidelines. These factors make them ideal case studies for assessing whether LLMs can provide accurate, guideline-adherent health information.

### Scoring and analysis of LLM responses

Two independent researchers and research assistants who were blinded to each other’s assessments, the specific LLM, the prompt, and its level of informedness scored the LLM responses using a validated scoring instrument, MAPPinfo^42^ for evidence-based health information quality, and, in addition to that, the proposal ebmNucleus, which draws on the guideline for evidence-based health information^3^ (Supplementary Table 3). Both tools share certain criteria (Table 3) but differ in their focus: while MAPPinfo provides a broad assessment of information quality, ebmNucleus concentrates on the core aspects of health decision-making, minimizing the influence of contextual factors irrelevant to most LLMs (e.g., authorship, graphical elements). Each LLM response was systematically labeled and scored accordingly.

**Table 3:** Criteria of the health information quality scoring instruments used in Study 2

MAPPinfo criteria	Overlapping criteria	ebmNucleus criteria
	“Informed decision”	
	Benefit numbers	
	Conflict of interest (COI)	
	Diagnostic quality	
	Evidence methodology	
	Final update	
	Harm numbers	
	No narratives	
	References	
	Stochastic uncertainty	
Authors		
Framing		-
Health problem		
COI management		
Natural course/prevalence		
Options		
Recipients’ definition		
Suitable graphics		
		Evidence quality
		Neutrality
		Patient-relevant benefits and harms
		Referencing single event probabilities
		Referral to support

Here, the evidence underlying the content was assessed according to the best available evidence (summarized under www.hardingcenter.de/en/transfer-and-impact/fact-boxes) from 2022, aligning with the training corpora of the LLMs used. While responses were consistently scored on a zero to two-point scale in MAPPinfo, responses concerning patient-relevant benefits and harms, referencing of single event probabilities, benefit and harm numbers, evidence quality, and interpretation of screening results was scored on a zero to three-point scale in ebmNucleus, while all other aspects were scored on a binary zero to one-point scale. Responses classified as ‘hallucinations’ or absurd statements were scored accordingly.

The raters were research assistants (RAs) and experienced researchers who had previously conducted similar coding tasks in multiple studies. The latter had expertise in evidence-based health communication. Each rater was instructed how to apply the scoring instruments. Two RA’s (each 50%) and one researcher independently scored LLM responses according to MAPPinfo; dyads from a pool of five RA’s independently scored according to ebmNucleus (Supplementary Table S3). The highest possible scoring (three points) of a test result interpretation required, for instance, providing the probability that a positive result may be due to underlying disease (=positive predictive value). The lowest possible score was applied when there was no hint on the limits of the screening test or just one-sided (false negatives).

Interrater reliability across all LLM outputs, calculated using Cohen’s kappa coefficient, was ϰ = 0.41 for MAPPinfo and ϰ = 0.38 for ebmNucleus. Because the reliability was too low, a third rater, a researcher (FGR and CW, respectively), who was blinded to the previous ratings, rated cases of disagreement. Their experienced ratings resulted each in ϰ = 0.94 agreement with the respective prior raters.

Raw sum scores were calculated for both MAPPinfo and ebmNucleus. Average sum scores were calculated for each LLM and prompt category and converted into the rate achieved regarding perfect evidence-based criteria. Descriptive statistics and variance analyses were conducted to examine the effect of prompting informedness on scoring outcomes.

### Study 2

Study 2 employed systematic investigations of laypeople’s prompting to assess the extent to which LLM-generated responses were evidence-based and how this could be improved through a boosting intervention. Reporting followed the STROBE^53^ and TRIPOD-LLM^54^ guidelines.

### Sample

Without prior evidence of the effect of a boost on LLM prompting, we focused on detecting a meaningful effect rather than any small difference. So, for detecting a moderate ANOVA main effect (based on Cohen’s^61^ convention for eta squared = 0.06, which equals partial eta squared for the one-way ANOVA) when comparing two between-subjects conditions (with and without intervention), we required a minimum sample size of *n* = 237 participants. To approximate simplified census data of Great Britain in terms of sex, age, and ethnicity, we recruited *n* = 300 adult participants from an online-representative pool of participants via Prolific. Their remuneration was €1.30 upon completion of the study.

### Design

We used a 2 × 3 between-subjects design: participants were randomly assigned either to standard prompting instructions (control) or enhanced prompting instructions (boosting intervention) and to one of three LLMs; OpenAI ChatGPT (gpt-3.5-turbo), Google Gemini (1.5-Flash), or Mistral AI Le Chat (mistral-large-2402). To ensure that participants could generate detailed prompts and receive more comprehensive responses, the survey was restricted to tablets and computers. Typing on smartphones is not equivalent to using a full keyboard, which could affect both the quality and complexity of the prompts as well as the resulting responses.

A minimal boosting intervention encouraged participants to consider the possible consequences of their choices as follows: “Please consider the OARS rule: You need to know your Options, the Advantages and Risks of each, and how Steady they are to happen.” In accordance with the competence principle of boosting^45^, the researchers developed the intervention on the basis of a prior shared decision making intervention for patients (“Ask three questions”^62^) and included it in a pre-test. Participants in the control group did not receive this intervention.

To ensure a balanced distribution of participants across study groups, we employed built-in block randomization (algorithmically managed, without fixed block size declarations) of the platform SoSci Survey where we hosted the study. Both participants and researchers were blinded to the assigned LLM and the type of prompting instructions to prevent bias in interaction and response evaluation.

The study was pre-registered with the German Clinical Trials Register (DRKS) (Registration No. DRKS00035228, registered on 15 October 2024) and received ethical approval from the Ethics Committee of the University of Potsdam (Approval No. 52/2024). It was conducted in October 2024 in accordance with the Declaration of Helsinki. All participants provided electronic informed consent.

### Pre-test

A pre-test that involved *n* = 20 participants was conducted to verify the effectiveness of the randomization procedures, test the technical functionality of the survey platform and its integration with the LLM APIs. Additionally, it measured the average completion time and identified potential issues related to participant burden. User feedback was collected to address any usability concerns, and necessary adjustments were made to optimize the study protocol for the main trial. The pre-test also assessed the study materials, particularly the clarity and comprehensibility of the survey questions and instructions.

### Procedure and material

Participants received a brief introduction outlining the study’s purpose, which involved interacting with one of three LLMs, all preset as a “helpful assistant” to obtain health information about either BC or PC screening. After giving informed consent, participants completed the questionnaire, providing basic demographic details such as gender, age, and education level. Participants could choose between BC and PC screening, regardless of their self-reported gender. This approach aimed to enhance engagement and data relevance while maintaining the integrity of the study design. They were then instructed to gather information on their chosen topic, which was expected to increase variance in information quality compared to focusing solely on mammography and PSA testing.

Participants interacted with the chatbot by entering their queries (prompts) into the questionnaire interface (Supplementary Fig. 1) that was connected via the LLM’s API, and the generated responses were collected alongside the prompts for systematic analysis in pre-defined internal variables in SoSci Survey. The interface allowed for multi-turn conversations: participants could submit follow-up questions within the same session, and the LLMs had access to the full chat history to generate context-sensitive responses. To ensure comparability across participants and maintain a controlled interaction length, each participant could submit a maximum of four prompts. At the end of the session, participants reported their frequency of LLM usage, prior experience with such models, and their attitude on shared decision-making. On average, participants completed the survey (Supplementary Table 4) in five minutes.

### Measures

In addition to demographic measures such as self-reported gender, age, and education level, we assessed LLM usage frequency, prior experience, and preferred approach to medical decision-making using specific items. LLM usage frequency was measured with the question, “How often have you used computer programs like ChatGPT (large language models) to get health information?” Response options included: Never, About 1–5 times per year, About 1–2 times per month, About 1–2 times per week, More frequently. Prior general experience was assessed with the item, “Please rate your experience with computer programs like ChatGPT (large language models).” Participants responded on a scale: Definitely no experience, Rather few experience, Some experience, Rather much experience, Definitely much experience. Their attitude on shared decision-making was elicited with statements about decision responsibility between GP and oneself.

### Scoring and analysis

To evaluate the responses generated by the LLMs, scoring followed the same procedure as in Study 1. Two raters independently (one RA, one researcher according to MAPPinfo; two researchers according to ebmNucleus) scored the 300 LLM outputs. Interrater reliability, calculated using Cohen’s kappa coefficient, was ϰ = 0.44 for MAPPinfo and ϰ = 0.71 for ebmNucleus. A third rater, a researcher (FGR or CW), who was blinded to the previous ratings, scored cases of disagreement. Their experienced ratings resulted in ϰ = 0.99 (MAPPinfo) and ϰ = 0.98 (ebmNucleus) agreement with the respective prior raters.

Raw sum scores were calculated for both MAPPinfo and ebmNucleus. The average sum scores were computed for each LLM and prompt category and converted into the rate of adherence to perfect evidence-based criteria. Descriptive statistics were calculated for demographic variables such as self-reported gender, age, and education, as well as for LLM usage frequency and prior experience. Variance analyses were conducted to examine the impact of prompt informedness on response quality, while interaction effects between LLM type and prompting instructions were also evaluated. Regression analyses explored the influence of demographic characteristics, LLM usage and prior experience.

All statistical tests were conducted at a significance level of *p* < 0.05, and effect sizes were calculated to determine the practical significance of findings.

## Supplementary information

Supplementary Information

## Data Availability

All data supporting the findings of this study are available from the authors.

## Code availability

The analysis code is available from the authors.
