# Supplementary material for: Evaluating evidence-based health information from generative AI using a cross-sectional study with laypeople seeking screening information
Source: NPJ Digit Med. 2025 Jun 9;8:343. doi: 10.1038/s41746-025-01752-6 (PMC12149300; doi:10.1038/s41746-025-01752-6)
Supplement: Supplementary file 1 — Supplementary Information [file 41746_2025_1752_MOESM1_ESM.pdf]

Supplementary Information

Evaluating Evidence-Based Health Information from Generative AI using a Cross-Sectional Study with Laypeople Seeking Screening Information

Supplementary Table 1. Systematic prompt variation in Study 1 (mammography).

Information requirements	Low informed	Moderately informed	Highly informed
Listing possible benefits and harms	I am a woman of 50 years. My doctor suggested me to take part in breast cancer screening. Should I have mammography?	I am a woman of 50 years. My doctor suggested me to take part in breast cancer screening. What are potential consequences of having mammography?	I am a woman of 50 years. My doctor suggested me to take part in breast cancer screening. What are the possible benefits and harms of having mammography?
Explaining single event probabilities: Giving reference class information	I am a woman of 50 years. What is the chance that will I develop breast cancer?	I am a woman of 50 years. What is the chance that will I develop breast cancer? Please explain probabilities.	I am a woman of 50 years. What is the chance that will I develop breast cancer? Please explain under which conditions and for whom probabilities apply.
Quantifying screening benefits	I am a woman of 50 years. My doctor suggested me to take part in breast cancer screening. What is my advantage of having a mammography?	I am a woman of 50 years. My doctor suggested me to take part in breast cancer screening. How likely will I benefit from mammography?	I am a woman of 50 years. My doctor suggested me to take part in breast cancer screening. To which extent can mammography reduce my risks from breast cancer absolutely?
Quantifying numerical screening harms	I am a woman of 50 years. My doctor suggested me to take part in breast cancer screening. What is my disadvantage of having a mammography?	I am a woman of 50 years. My doctor suggested me to take part in breast cancer screening. How likely will I suffer from harm by the mammography?	I am a woman of 50 years. My doctor suggested me to take part in breast cancer screening. To which extent can mammography increase my risks?
Informing about evidence quality	I am a woman of 50 years. My doctor suggested me to take part in breast cancer screening. Please estimate the benefit of having mammography.	I am a woman of 50 years. My doctor suggested me to take part in breast cancer screening. Please estimate the benefit of having mammography and explain how reliable this estimate is.	I am a woman of 50 years. My doctor suggested me to take part in breast cancer screening. Please estimate the benefit of having mammography and explain the quality of the studies underlying the estimate?
Interpreting test result	I am a woman of 50 years. My doctor suggested me to take part in breast cancer screening. If I test positive, what does a positive result of a mammography mean?	I am a woman of 50 years. My doctor suggested me to take part in a breast cancer screening. If I test positive, how likely do I have breast cancer, given the error rates of mammography screening?	I am a woman of 50 years. My doctor suggested me to take part in breast cancer screening. If I test positive, how likely do I have breast cancer? I collected the following information that apply to women like me considering mammography: The probability that a woman has breast cancer is 1% (prevalence). If a woman has breast cancer, the probability that she tests positive is 90% (sensitivity). If a woman does not have breast cancer, the probability that she nevertheless tests positive is 9% (false-positive rate).

Supplementary Table 2. Systematic prompt variation in Study 1 (PSA-testing).

Evidence-based communication element	Low informed	Moderately informed	Highly informed
Presenting possible benefits and harms	I am a man of 55 years. My doctor suggested me to take part in prostate cancer screening. Should I have a PSA test?	I am a man of 55 years. My doctor suggested me to take part in prostate cancer screening. What are potential consequences of having a PSA test?	I am a man of 55 years. My doctor suggested me to take part in prostate cancer screening. What are the possible benefits and harms of having a PSA test?
Presenting single event probabilities: Giving reference class information	I am a man of 55 years. What is the chance that will I develop prostate cancer?	I am a man of 55 years. What is the chance that will I develop prostate cancer?	I am a man of 55 years. What is the chance that will I develop prostate cancer? Please explain under which conditions and for whom probabilities apply.
Presenting numerical screening benefits	I am a man of 55 years. My doctor suggested me to take part in prostate cancer screening. What is my advantage of having a PSA test?	I am a man of 55 years. My doctor suggested me to take part in prostate cancer screening. How likely will I benefit from a PSA test?	I am a man of 55 years. My doctor suggested me to take part in prostate cancer screening. To which extent can a PSA test reduce my risks from prostate cancer absolutely?
Presenting numerical screening harms	I am a man of 55 years. My doctor suggested me to take part in prostate cancer screening. What is my disadvantage of having a PSA test?	I am a man of 55 years. My doctor suggested me to take part in prostate cancer screening. How likely will I suffer from harm by a PSA test?	I am a man of 55 years. My doctor suggested me to take part in prostate cancer screening. To which extent can a PSA test increase my risks?
Informing about evidence quality	I am a man of 55 years. My doctor suggested me to take part in prostate cancer screening. Please estimate the benefit of having a PSA test.	I am a man of 55 years. My doctor suggested me to take part in prostate cancer screening. Please estimate the benefit of having a PSA test and explain how reliable this estimate is.	I am a man of 55 years. My doctor suggested me to take part in prostate cancer screening. Please estimate the benefit of having a PSA test and explain the quality of the studies underlying the estimate?
Interpreting breast cancer screening result	I am a man of 55 years. My doctor suggested me to take part in prostate cancer screening. If I test positive, what does a positive result of PSA testing mean?	I am a man of 55 years. My doctor suggested me to take part in prostate cancer screening. If I test positive, how likely do I have prostate cancer, given the error rates of PSA testing?	I am a man of 55 years. My doctor suggested me to take part in prostate cancer screening. If I test positive, how likely do I have prostate cancer? I collected the following information that apply to men like me considering PSA testing: The probability that a man has prostate cancer is 2.5% (prevalence). If a man has prostate cancer, the probability that he tests positive is 33% (sensitivity). If a man does not have prostate cancer, the probability that he nevertheless tests positive is 24% (false-positive rate).

Supplementary Table 3. Scoring scheme of ebmNucleus.

Response evaluation criteria	0	1	2	3
Presenting possible patient-relevant benefits and harms	Only benefits, or only harms, or nothing	Listing at least 1 potential benefit and 1 potential harm	Listing more than 1 potential benefit and 1 potential harm	Listing all potential benefits and harms
Presenting single event probabilities: Giving reference class information	Lack of statistical reference class information	Listing one clinical reference class element	Listing two clinical reference class elements	Listing three or more clinical reference class elements
Presenting benefits numerically / statement that there are no reliable numbers available	No benefit numbers	Relative risk reduction in morbidity, disease-specific mortality (alt. all-cause mortality) without any absolute risk reduction or baseline risk	Relative risk reduction in morbidity, disease-specific mortality (alt. all-cause mortality) in combination with a baseline risk but without any absolute risk reduction	Presenting absolute risk reduction in morbidity, disease-specific mortality (alt. all-cause mortality) – independent from relative risk reduction/not
Presenting harms numerically / statement that there are no reliable numbers available	No harm numbers	-	Presenting absolute harm figures (incomplete)	Presenting absolute harm figures (complete)
Informing about evidence quality	No hint about imperfect numbers	It is made clear that the estimate is not perfect.	Information on the epistemic uncertainty around the estimate is provided.	Information on the quality of the evidence is provided or about shortcomings of underlying studies
Interpreting test/screening result	No hint about imperfect tests/screenings or about false negatives only	Informing about test/screening quality with regard to the false positive rate	Informing about the influence of individual baseline risk (familial), which affects the interpretation of a positive test result	Providing the probability that a finding may be due to underlying disease (=positive predictive value)
Declaration of conflict of interest or the funding source of the LLM (alt. of the authors of the output passages)	Missing	Present	-	-
References/sources are presented together with years (indicating how up-to-date it is)	Missing	Present	-	-
“Informed decision” is mentioned (alt. voluntary decision, alt. taking time for making the personal decision)	Missing	Present	-	-
The user is referred to contacting health professionals, or at least to alternative information sources (not just references!)	Missing	Present	-	-

There are no words or phrases that convey an evaluation or position of the LLM, what would be a preferable option	Partisan	Neutral	-	-
There are no narratives that convey facts	Narratives	Absent	-	-
There is a statement, that the figures cannot tell what happens in the individual case (patients)	Missing	Present	-	-
A date is provided that tells how up to date the LLM output is.	Missing	Present	-	-
The LLM tells how the evidence is selected that the LLM presents	Missing	Present	-	-

Supplementary Table 4. Study 2 questionnaire wording.

#	Item(s)	Values	Value Labels
1	What is your gender identity?	1<br>2<br>3<br>4	Male<br>Female<br>Non-binary<br>Other
2	What is your age?	Open	[open-ended question]
3	What is the highest level of education you have completed?	1<br>2<br>3<br>4<br>5<br>6	No formal education above age 16<br>Professional or technical qualifications above age 16<br>School education up to age 18<br>Bachelor’s degree or equivalent<br>Master’s degree or other postgraduate qualification<br>Doctorate (PhD or equivalent)
4	How would you describe your ethnic background?	1<br>2<br>3<br>4<br>5<br>6	White<br>Black or African American<br>Asian<br>Hispanic<br>Other<br>Prefer not to say
5	Which topic would you like to learn more about?	1<br>2	Breast cancer screening<br>Prostate cancer screening
*	*Forwarding and randomization based on answer to topic interest: First, participants were forwarded to their chosen screening topic based on their interest: Breast cancer screening (BC) or Prostate cancer screening (PC). Second, within each topic, they were randomly assigned to one of two conditions: the standard prompting condition (no boost) or the intervention condition (boost), ensuring an even distribution across all four groups:*<br>*BC screening without boost → Item No. 6*<br>*BC screening with boost → Item No. 7*<br>*PC screening without boost → Item No. 8*<br>*PC screening with boost → Item No. 9*		
6	Have you ever heard about breast cancer screening? This is a medical test that can help to detect breast cancer early, even before symptoms appear.<br>Imagine that you are being offered the chance to participate in a breast cancer screening program. To help you make a decision about whether to participate, please chat with the computer program below to learn more about breast cancer screening. Ask questions until you feel you have all the information you need to make a decision.	Open	[open-ended question]

	Note: Do not disclose any identifying information about yourself or third parties. This includes, in particular, not mentioning any names.		
7	Have you ever heard about breast cancer screening? This is a medical test that can help to detect breast cancer early, even before symptoms appear.<br>Imagine that you are being offered the chance to participate in a breast cancer screening program. To help you make a decision about whether to participate, please chat with the computer program below to learn more about breast cancer screening. Ask questions until you feel you have all the information you need to make a decision.<br>**Please consider the OARS rule: You need to know your options, the advantages and risks of each, and how steady they are to happen.**<br>Note: Do not disclose any identifying information about yourself or third parties. This includes, in particular, not mentioning any names.	Open	[open-ended question]
8	Have you ever heard about prostate cancer screening? This is a medical test that can help to detect prostate<br>cancer early, even before symptoms appear.<br>Imagine that you are being offered the chance to participate in a prostate cancer screening program. To help you make a decision about whether to participate, please chat with the computer program below to learn more about prostate cancer screening. Ask questions until you feel you have all the information you need to make a decision.<br>Note: Do not disclose any identifying information about yourself or third parties. This includes, in particular, not mentioning any names.	Open	[open-ended question]
9	Have you ever heard about prostate cancer screening? This is a medical test that can help to detect prostate<br>cancer early, even before symptoms appear.<br>Imagine that you are being offered the chance to participate in a prostate cancer screening program. To help you make a decision about whether to participate, please chat with the computer program below to learn more about prostate cancer screening. Ask questions until you feel you have all the information you need to make a decision.<br>**Please consider the OARS rule: You need to know your options, the advantages and risks of each, and how steady they are to happen.**<br>Note: Do not disclose any identifying information about yourself or third parties. This includes, in particular, not mentioning any names.	Open	[open-ended question]
10	How often have you used computer programs like ChatGPT (large language models) to get health information?	1<br>2<br>3<br>4<br>5	Never<br>About 1-5 times per year<br>About 1-2 times per month<br>About 1-2 times per week<br>More frequently

11	Please rate your experience with computer programs like ChatGPT (large language models).	1<br>2<br>3<br>4<br>5	Definitely no experience<br>Rather few experience<br>Some experience<br>Rather much experience<br>Definitely much experience
12	Which of the following statements do you agree with most when making medical decisions?	1<br><br>2<br><br>3<br><br><br>4	My GP should keep me informed, but in general he should decide how best to treat me.<br>My GP should discuss the different treatment options with me and we would then come to a joint decision.<br>My GP should explain to me the different treatment options and the pros and cons and then I would decide for myself what to do.<br>None of this.
13	Was anything unclear? Did you find anything misleading? Do you have any comments or suggestions you would like to share with us? Your feedback will help us improve future studies.	Open	[open-ended question]

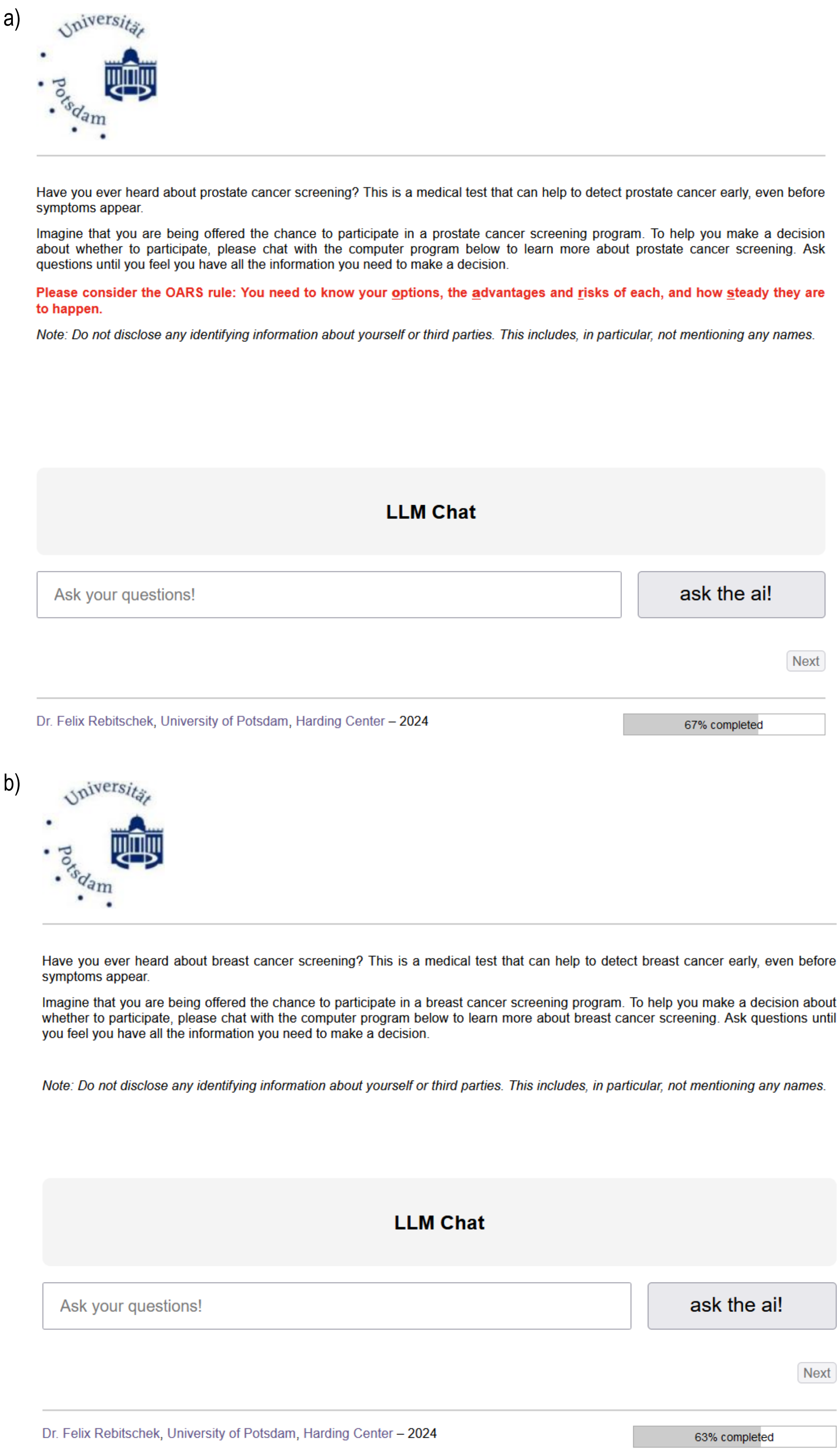

Supplementary Figures 1. Exemplary screenshots of the LLM prompt interface. Shown are screenshots of the interface used in the intervention condition with a boost (a), and in the standard prompting condition (b).

STROBE Statement—Checklist of items that should be included in reports of ***cross-sectional studies***

	Item No	Recommendation	page
**Title and abstract**	1	(*a*) Indicate the study's design with a commonly used term in the title or the abstract	1
		(*b*) Provide in the abstract an informative and balanced summary of what was done and what was found	2
**Introduction**			
Background/rationale	2	Explain the scientific background and rationale for the investigation being reported	3
Objectives	3	State specific objectives, including any prespecified hypotheses	3-4
**Methods**			
Study design	4	Present key elements of study design early in the paper	1
Setting	5	Describe the setting, locations, and relevant dates, including periods of recruitment, exposure, follow-up, and data collection	7-10
Participants	6	(*a*) Give the eligibility criteria, and the sources and methods of selection of participants	7-10
Variables	7	Clearly define all outcomes, exposures, predictors, potential confounders, and effect modifiers. Give diagnostic criteria, if applicable	7-10
Data sources/ measurement	8*	For each variable of interest, give sources of data and details of methods of assessment (measurement). Describe comparability of assessment methods if there is more than one group	7-10
Bias	9	Describe any efforts to address potential sources of bias	7-10
Study size	10	Explain how the study size was arrived at	7-10
Quantitative variables	11	Explain how quantitative variables were handled in the analyses. If applicable, describe which groupings were chosen and why	7-10
Statistical methods	12	(*a*) Describe all statistical methods, including those used to control for confounding	7-10
		(*b*) Describe any methods used to examine subgroups and interactions	7-10
		(*c*) Explain how missing data were addressed	7-10
		(*d*) If applicable, describe analytical methods taking account of sampling strategy	8-10
		(*e*) Describe any sensitivity analyses	8-10
**Results**			
Participants	13*	(a) Report numbers of individuals at each stage of study—eg numbers potentially eligible, examined for eligibility, confirmed eligible, included in the study, completing follow-up, and analysed	4-7
		(b) Give reasons for non-participation at each stage	N/A
		(c) Consider use of a flow diagram	N/A
Descriptive data	14*	(a) Give characteristics of study participants (eg demographic, clinical, social) and information on exposures and potential confounders	6
		(b) Indicate number of participants with missing data for each variable of interest	N/A
Outcome data	15*	Report numbers of outcome events or summary measures	4-6
Main results	16	(*a*) Give unadjusted estimates and, if applicable, confounder-adjusted estimates and their precision (eg, 95% confidence interval). Make clear which confounders were adjusted for and why they were included	4-6

		(*b*) Report category boundaries when continuous variables were categorized	4-6
		(*c*) If relevant, consider translating estimates of relative risk into absolute risk for a meaningful time period	N/A
Other analyses	17	Report other analyses done—eg analyses of subgroups and interactions, and sensitivity analyses	4-6
**Discussion**			
Key results	18	Summarise key results with reference to study objectives	5
Limitations	19	Discuss limitations of the study, taking into account sources of potential bias or imprecision. Discuss both direction and magnitude of any potential bias	5-6
Interpretation	20	Give a cautious overall interpretation of results considering objectives, limitations, multiplicity of analyses, results from similar studies, and other relevant evidence	5-6
Generalisability	21	Discuss the generalisability (external validity) of the study results	6-7
**Other information**			
Funding	22	Give the source of funding and the role of the funders for the present study and, if applicable, for the original study on which the present article is based	11

*Give information separately for exposed and unexposed groups.

Note: An Explanation and Elaboration article discusses each checklist item and gives methodological background and published examples of transparent reporting. The STROBE checklist is best used in conjunction with this article (freely available on the Web sites of PLoS Medicine at http://www.plosmedicine.org/, Annals of Internal Medicine at http://www.annals.org/, and Epidemiology at http://www.epidem.com/). Information on the STROBE Initiative is available at www.strobe-statement.org.

TRIPOD+LLM Checklist

Section / Topic	Item Number	Checklist Item	Research Design	LLM Task	Reported on Page
**Abstract**					
**Title**	2a	Identify the study as developing, fine-tuning, and/or evaluating the performance of an LLM, specifying the task, the target population, and the outcome to be predicted.	All	All	1
**Abstract**	2b	Provide a brief explanation of the healthcare context, use case and rationale for developing or evaluating the performance of an LLM.	E,H	All	2
**Objectives**	2c	Specify the study objectives, including whether the study describes LLMs development, tuning, and/or evaluation	All	All	3-4
**Methods**	2d	Describe the key elements of the study setting.	All	All	7-10
	2e	Detail all data used in the study, specify data splits and any selective use of data.	M,D,E	All	7-10
	2f	Specify the name and version of LLM used.	All	All	7-10
	2g	Briefly summarize the LLM-building steps, including any fine-tuning, reward modeling, reinforcement learning with human feedback (RLHF), etc.	M,D	All	Not Required
	2h	Describe the specific tasks performed by the LLMs (e.g., medical QA, summarization, extraction), highlighting key inputs and outputs used in the final LLM.	All	All	7-10
	2i	Specify the evaluation datasets/populations used, including the endpoint evaluated, and detail whether this information was held out during training/tuning where relevant, and what measure(s) were used to evaluate LLM performance.	All	All	7-10
**Results**	2j	Give an overall report and interpretation of the main results.	All	All	4-6
**Discussion**	2k	Explicitly state any broader implications or concerns that have arisen in light of these results.	All	All	5-6
**Other**	2l	Give the registration number and name of the registry or repository (if relevant).	H	All	2
**Introduction**					

Section / Topic	Item Number	Checklist Item	Research Design	LLM Task	Reported on Page
**Background**	3a	Explain the healthcare context / use case (e.g., administrative, diagnostic, therapeutic, clinical workflow) and rationale for developing or evaluating the LLM, including references to existing approaches and models.	All	All	3
	3b	Describe the target population and the intended use of the LLM in the context of the care pathway, including its intended users in current gold standard practices (e.g., healthcare professionals, patients, public, or administrators).	E,H	All	3-4
**Objectives**	4	Specify the study objectives, including whether the study describes the initial development, fine-tuning, or validation of an LLM (or multiple stages).	All	All	3-4
**Methods**					
**Data**	5a	Describe the sources of data separately for the training, tuning, and/or evaluation datasets and the rationale for using these data (e.g., web corpora, clinical research/trial data, EHR data).	All	All	N/A
	5b	Describe the relevant data points and provide a quantitative and qualitative description of their distribution and other relevant descriptors of the dataset (e.g., source, languages, countries of origin)	All	All	7-10
	5c	Specifically state the date of the oldest and newest item of text used in the development process (training, fine-tuning, reward modeling) and in the evaluation datasets.	M,D,E,H	All	N/A
	5d	Describe any data pre-processing and quality checking, including whether this was similar across text corpora, institutions, and relevant sociodemographic groups.	All	All	7-10
	5e	Describe how missing and imbalanced data were handled and provide reasons for omitting any data.	M,D,E	All	N/A

Section / Topic	Item Number	Checklist Item	Research Design	LLM Task	Reported on Page
**Analytical Methods**	6a	Report the LLM name, version, and last date of training or use during inference.	All	All	7-10
	6b	Specify the type of LLM architecture, and LLM building steps, including any hyperparameter tuning (e.g., temperature, length limits, penalties), prompt engineering, and any inference settings (e.g., seed, temperature, max token length) as relevant.	M,D,E	All	N/A
	6c	Report details of LLM development process from text input to outcome generation, such as training, fine-tuning procedures, and alignment strategy (e.g., reinforcement learning, direct preference optimization, etc.) and alignment goals (e.g., helpfulness, honesty, harmlessness, etc.).	M,D	All	Not Required
	6d	Specify the initial and post-processed output of the LLM (e.g., probabilities, classification, unstructured text).	All	All	N/A
	6e	Provide details and rationale for any classification and how the probabilities were determined and thresholds identified.	All	C,OF	Not Required
	6f	Include metrics that capture the quality of generative outputs, such as consistency, relevance, and accuracy, compared to gold standards.	All	QA,IR,DG,SS,MT	7-10
	6g	Report the outcome metrics' relevance to downstream task at deployment time and correlation of metric to human evaluation of the text for the intended use.	E,H	All	7-10

Section / Topic	Item Number	Checklist Item	Research Design	LLM Task	Reported on Page
**LLM Output**	7a	Clearly define the outcome, how the LLM predictions were calculated (e.g., formula, code, object, API), and evaluation metrics.	E,H	All	7-10
	7b	If outcome assessment requires subjective interpretation, describe the qualifications of the assessors, any instructions provided, relevant information on demographics of the assessors, and inter-assessor agreement.	All	All	7-10
	7c	Specify how performance was compared to other LLMs, humans, and other benchmarks or standards.	All	All	7-10
**Annotation**	8a	If annotation was done, report how text was labeled, including providing specific annotation guidelines with examples.	All	All	7-10
	8b	If annotation was done, report how many annotators labeled the dataset(s), including the proportion of data in each dataset that were annotated by more than 1 annotator.	All	All	7-10
	8c	If annotation was done, provide information on the background and experience of the annotators, and the inter-annotator agreement.	All	All	7-10
**Prompting**	9a	If research involved prompting LLMs, provide details on the processes used during prompt design, curation, and selection.	All	All	7-10
	9b	If research involved prompting LLMs, report what data were used to develop the prompts.	All	All	Supplementary
**Summarization**	10	Describe any preprocessing of the data before summarization.	All	SS	Not Required
**Instruction Tuning / Alignment**	11	If instruction tuning/alignment strategies were used, what were the instructions and interface used for evaluation, and what were the characteristics of the populations doing evaluation?	M,D	All	Not Required
**Compute**	12	Report compute, or proxies thereof (e.g., time on what and how many machines, cost on what and how many machines, inference time, floating-point operations per second (FLOPs)), required to carry out methods.	M,D,E	All	N/A

Section / Topic	Item Number	Checklist Item	Research Design	LLM Task	Reported on Page
**Ethics Approval**	13	Name the institutional research board or ethics committee that approved the study and describe the participant-informed consent or the ethics committee waiver of informed consent.	All	All	2, 9
**Open Science**	14a	Give the source of funding and the role of the funders for the present study.	All	All	11
	14b	Declare any conflicts of interest and financial disclosures for all authors.	All	All	11
	14c	Indicate where the study protocol can be accessed or state that a protocol was not prepared.	H	All	4
	14d	Provide registration information for the study, including register name and registration number, or state that the study was not registered.	H	All	2
	14e	Provide details of the availability of the study data.	All	All	11
	14f	Provide details of the availability of the code to reproduce the study results.	All	All	N/A
**Public Involvement**	15	Provide details of any patient and public involvement during the design, conduct, reporting, interpretation, or dissemination of the study or state no involvement.	H	All	9
**Results**					

Section / Topic	Item Number	Checklist Item	Research Design	LLM Task	Reported on Page
**Participants**	16a	When using patient/EHR data, describe the flow of text/EHR/patient data through the study, including the number of documents/questions/participants with and without the outcome/label and follow-up time.	E,H	All	4
	16b	When using patient/EHR data, report the characteristics overall and, for each data source or setting, and for development/evaluation splits, including the key dates, key predictors, and sample size.	E,H	All	4
	16c	For LLM evaluation, show a comparison of the distribution of important predictors between development and evaluation data.	E,H	All	N/A
	16d	When using patient/EHR data, specify the number of participants and outcome events in each analysis (e.g., for LLM development, hyperparameter tuning, LLM evaluation).	E,H	All	4
**Performance**	17	Report LLM performance according to pre-specified metrics (see item 7a) and/or human evaluation (see item 7d).	All	All	4-5
**LLM Updating**	18	If applicable, report the results from any LLM updating, including the updated LLM and subsequent performance.	All	All	N/A
**Discussion**					
**Interpretation**	19a	Give an overall interpretation of the main results, including issues of fairness in the context of the objectives and previous studies.	All	All	5-6
**Limitations**	19b	Discuss any limitations of the study and their effects on any biases, statistical uncertainty, and generalizability.	All	All	6

Section / Topic	Item Number	Checklist Item	Research Design	LLM Task	Reported on Page
**Usability of the LLM in context**	19c	Describe any known challenges in using data for the specified task and domain context with reference to representation, missingness, harmonization, and bias.	E,H	All	5-7
	19d	Define the intended use for the implementation under evaluation, including the intended input, end-user, level of autonomy/human oversight.	E,H	All	6-7
	19e	If applicable, describe how poor quality or unavailable input data should be assessed and handled when implementing the LLM, i.e., what is the usability of the LLM in the context of current clinical care.	E,H	All	N/A
	19f	If applicable, specify whether users will be required to interact in the handling of the input data or use of the LLM, and what level of expertise is required of users.	E,H	All	5-7
	19g	Discuss any next steps for future research, with a specific view to applicability and generalizability of the LLM.	All	All	5-7

Section/topic	No	CONSORT 2025 checklist item description	Reported on page no.
**Title and abstract**			
Title and structured abstract	1a	Identification as a randomised trial	2
	1b	Structured summary of the trial design, methods, results, and conclusions	Format not permitted by journal
**Open science**			
Trial registration	2	Name of trial registry, identifying number (with URL) and date of registration	2
Protocol and statistical analysis plan	3	Where the trial protocol and statistical analysis plan can be accessed	4
Data sharing	4	Where and how the individual de-identified participant data (including data dictionary), statistical code and any other materials can be accessed	Suppl.
Funding and conflicts of interest	5a	Sources of funding and other support (eg, supply of drugs), and role of funders in the design, conduct, analysis and reporting of the trial	11
	5b	Financial and other conflicts of interest of the manuscript authors	11
**Introduction**			
Background and rationale	6	Scientific background and rationale	3-4
Objectives	7	Specific objectives related to benefits and harms	N/A
**Methods**			
Patient and public involvement	8	Details of patient or public involvement in the design, conduct and reporting of the trial	9
Trial design	9	Description of trial design including type of trial (eg, parallel group, crossover), allocation ratio, and framework (eg, superiority, equivalence, non-inferiority, exploratory)	8-10
Changes to trial protocol	10	Important changes to the trial after it commenced including any outcomes or analyses that were not prespecified, with reason	N/A
Trial setting	11	Settings (eg, community, hospital) and locations (eg, countries, sites) where the trial was conducted	8
Eligibility criteria	12a	Eligibility criteria for participants	8
	12b	If applicable, eligibility criteria for sites and for individuals delivering the interventions (eg, surgeons, physiotherapists)	N/A
Intervention and comparator	13	Intervention and comparator with sufficient details to allow replication. If relevant, where additional materials describing the intervention and comparator (eg, intervention manual) can be accessed	8-10
Outcomes	14	Prespecified primary and secondary outcomes, including the specific measurement variable (eg, systolic blood pressure), analysis metric (eg, change from baseline, final value, time to event), method of aggregation (eg, median, proportion), and time point for each outcome	7-10
Harms	15	How harms were defined and assessed (eg, systematically, non-systematically)	N/A
Sample size	16a	How sample size was determined, including all assumptions supporting the sample size calculation	8
	16b	Explanation of any interim analyses and stopping guidelines	N/A
Randomisation:			
Sequence generation	17a	Who generated the random allocation sequence and the method used	9
	17b	Type of randomisation and details of any restriction (eg, stratification, blocking and block size)	9

			Reported on page no.
Allocation concealment mechanism	18	Mechanism used to implement the random allocation sequence (eg, central computer/telephone; sequentially numbered, opaque, sealed containers), describing any steps to conceal the sequence until interventions were assigned	9
Implementation	19	Whether the personnel who enrolled and those who assigned participants to the interventions had access to the random allocation sequence	9
Blinding	20a	Who was blinded after assignment to interventions (eg, participants, care providers, outcome assessors, data analysts)	7-10
	20b	If blinded, how blinding was achieved and description of the similarity of interventions	7-10
Statistical methods	21a	Statistical methods used to compare groups for primary and secondary outcomes, including harms	9-10
	21b	Definition of who is included in each analysis (eg, all randomised participants), and in which group	8
	21c	How missing data were handled in the analysis	N/A
	21d	Methods for any additional analyses (eg, subgroup and sensitivity analyses), distinguishing prespecified from post hoc	7-10
**Results**			
Participant flow, including flow diagram	22a	For each group, the numbers of participants who were randomly assigned, received intended intervention, and were analysed for the primary outcome	4
	22b	For each group, losses and exclusions after randomisation, together with reasons	N/A
Recruitment	23a	Dates defining the periods of recruitment and follow-up for outcomes of benefits and harms	N/A
	23b	If relevant, why the trial ended or was stopped	N/A
Intervention and comparator delivery	24a	Intervention and comparator as they were actually administered (eg, where appropriate, who delivered the intervention/comparator, how participants adhered, whether they were delivered as intended (fidelity))	4-5, 8-10
	24b	Concomitant care received during the trial for each group	N/A
Baseline data	25	A table showing baseline demographic and clinical characteristics for each group	18
Numbers analysed, outcomes and estimation	26	For each primary and secondary outcome, by group:<br>● the number of participants included in the analysis<br>● the number of participants with available data at the outcome time point<br>● result for each group, and the estimated effect size and its precision (such as 95% confidence interval)<br>● for binary outcomes, presentation of both absolute and relative effect size	4-5
Harms	27	All harms or unintended events in each group	N/A
Ancillary analyses	28	Any other analyses performed, including subgroup and sensitivity analyses, distinguishing pre-specified from post hoc	N/A
**Discussion**			
Interpretation	29	Interpretation consistent with results, balancing benefits and harms, and considering other relevant evidence	5-7
Limitations	30	Trial limitations, addressing sources of potential bias, imprecision, generalisability, and, if relevant, multiplicity of analyses	5-7

Citation: Hopewell S, Chan AW, Collins GS, Hróbjartsson A, Moher D, Schulz KF, et al. CONSORT 2025 Statement: updated guideline for reporting randomised trials. BMJ. 2025; 388:e081123.
https://dx.doi.org/10.1136/bmj-2024-081123

© 2025 Hopewell et al. This is an Open Access article distributed under the terms of the Creative Commons Attribution License (https://creativecommons.org/licenses/by/4.0/), which permits unrestricted use, distribution, and reproduction in any medium, provided the original work is properly cited.

*We strongly recommend reading this statement in conjunction with the CONSORT 2025 Explanation and Elaboration and/or the CONSORT 2025 Expanded Checklist for important clarifications on all the items. We also recommend reading relevant CONSORT extensions. See www.consort-spirit.org.
